# Criteria for Assessing Sustainability of Lignocellulosic Wastes: Applied to the Cellulose Nanofibril Packaging Production in the UK

**DOI:** 10.3390/polym15061336

**Published:** 2023-03-07

**Authors:** Samantha Islam, Jonathan M. Cullen

**Affiliations:** Department of Engineering, University of Cambridge, Trumpington Street, Cambridge CB2 1PZ, UK

**Keywords:** biodegradable packaging, lignocellulose, cellulose nanofibrils, feedstock selection, sustainability assessment, waste management

## Abstract

Extensive use of petrochemical plastic packaging leads to the greenhouse gas emission and contamination to soil and oceans, posing major threats to the ecosystem. The packaging needs, hence, are shifting to bioplastics with natural degradability. Lignocellulose, the biomass from forest and agriculture, can produce cellulose nanofibrils (CNF), a biodegradable material with acceptable functional properties, that can make packaging among other products. Compared to primary sources, CNF extracted from lignocellulosic wastes reduces the feedstock cost without causing an extension to agriculture and associated emissions. Most of these low value feedstocks go to alternative applications, making their use in CNF packaging competitive. To transfer the waste materials from current practices to the packaging production, it is imperative to assess their sustainability, encompassing environmental and economic impacts along with the feedstock physical and chemical properties. A combined overview of these criteria is absent in the literature. This study consolidates thirteen attributes, delineating sustainability of lignocellulosic wastes for commercial CNF packaging production. These criteria data are gathered for the UK waste streams, and transformed into a quantitative matrix, evaluating the waste feedstock sustainability for CNF packaging production. The presented approach can be adopted to decision scenarios in bioplastics packaging conversion and waste management.

## 1. Introduction

Plastics, the fossil-derived polymers, with strength, flexibility and durability, have wide range of applications, including packaging [1]. Packaging holds the largest global plastic market, presenting 36% of the overall demand in 2021 [2]. Most of the plastic packaging are single use and often end up in incineration or landfilling, causing major global greenhouse gas (GHG) emissions [1,3]. According to OECD [4], plastic life cycle globally accounts for 1.8 Gt CO_2_-equivalent emissions in 2019, which is projected to grow to 4.3 Gt by 2060. When proper disposal does not take place, plastics often enter the terrestrial and marine environments, negatively impacting the ecosystems for thousands of years, due to being non-biodegradable [5,6].

These prevalent environmental impacts have led to the shift of packaging consumption towards bioplastics, derived from biological precursors (e.g., starch, cellulose, alginate, gelatin, collagen, proteins, chitosan, pectin) with natural biodegradability [3,7,8]. The global bioplastics production capacity standing at 2.42 mtonnes in 2021 is projected to grow to 7.59 mtonnes in 2026 [9]. Starch-blends derived from food crops (e.g., maize, sugarcane) dominate the commercial market of bioplastic feedstocks [10,11] but present a number of problems. Consumption of food crop feedstocks threatens food security, increasing both the market demand and price [12]. This also increases the use of land and fertiliser with associated GHG emissions, negating the sought environmental benefits of bioplastics [13,14]. Moreover, material characteristics, e.g., poor mechanical and barrier properties of starch-based bioplastics make them an inferior alternative to their petrochemical counterparts [15,16].

**Figure 1:**
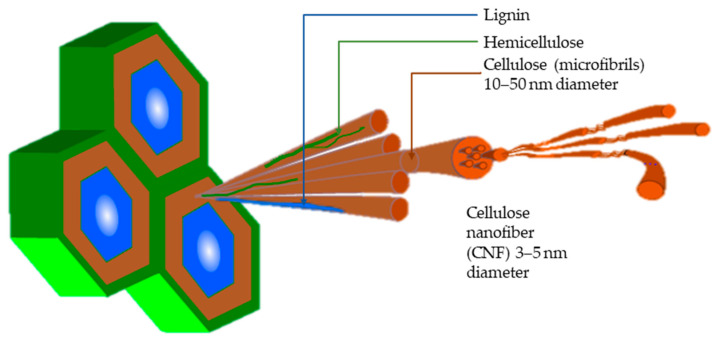
Hierarchical structure of cellulose fibres in LC cell wall.

Whereas, lignocellulose (LC), the biomass composites of cellulose, hemicellulose and lignin, deriving from forestry and agriculture, does not compete with food and are abundant in nature [17]. Some potential LC sources include: wood (softwood, hardwood), seed (cotton), bast (flax, hemp), leaf (sisal, brassica), stalk (wheat, barley) and grass/weed (miscanthus, Arabidopsis, bamboo) [18]. The cellulose fibres in LC are composed of microfibrils of 10–50 nm diameter, that in turn is comprised of elementary fibrils with a diameter of 3–5 nm, each of which consist of around 30–100 cellulose polymer chains (see Figure 1) [19]. Biosynthesis of LC can produce native nanocellulose materials: weblike cellulose nano fibrils (CNFs) and rodlike cellulose nanocrystals (CNCs) [17]. CNF gels, with larger surface areas, possess better film formation capability than CNC, and are therefore recommended for packaging applications [8,20].

Numerous studies discuss the use of CNF packaging films for: food, health care and various consumer goods [8,16,21]. These films are not only biodegradable and recyclable, but also demonstrate functionalities better or comparable to that of petrochemical polymers and other LC derivatives, e.g., regular paper [22,23]. CNF films demonstrate high mechanical strength, optical transmittance, thermal stability and gas (e.g., oxygen, air) barrier properties [24,25]. They also show better water vapor barrier properties than paper, though that remains somewhat lower compared to petrochemical plastics (e.g., polyolefins) [23]. This limits their application for packaging products with high moisture content (e.g., horticulture, fish, meat) and/or being stored at high relative humidity. However, this shortcoming could be overcome by various processes: incorporation of inorganic fillers (i.e., silver), chemical modification (i.e., plasma polymerization) and adsorption of other film matrix materials (e.g., chitosan) [26,27].

**Figure 2:**
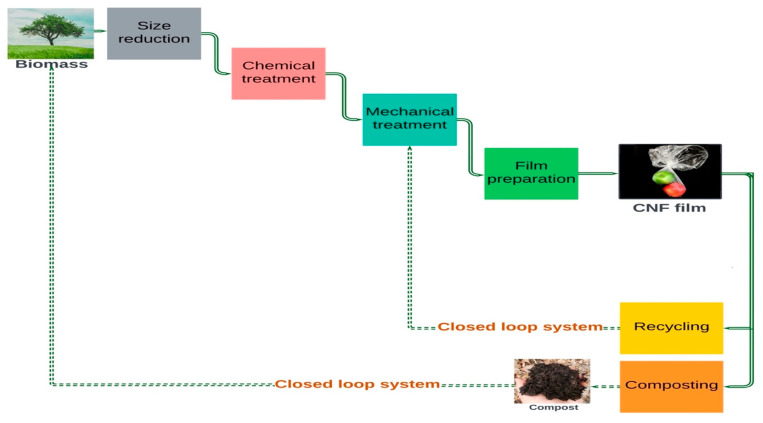
Generic CNF film processing from LC and end-of-life treatments.

CNF packaging films are produced mainly in four generic steps (See Figure 2): (1) size reduction, e.g., chopping or grinding of LCs; (2) chemical/biological pre-treatment for removal of non-cellulosic compounds (e.g., lignin, hemicellulose) or modifying properties; (3) mechanical disintegration of cellulose; and (4) film preparation [17,28]. The CNF films can be either recycled or converted into compost, returning organic matter to the soil [29,30]. Ease of preparation, competitive properties and circular end-of-life treatments spur commercial interests in CNF packaging production [26,29]. Large-scale production ought to fulfil a major proportion of the global demand for flexible packaging that stood at 33.5 million metric tons in 2022 [31,32].

Economic and environmental consequences are major obstacles for large-scale biodegradable packaging production [33,34]. Production of dedicated LC feedstock (i.e., purposefully cultivated for bioplastics) can lead to the land use changes as well as enhanced agricultural activities and fertilizer uses, causing a massive environmental burden [12,35,36]. Moreover, feedstock price, a major contributor to the LC processing cost, is higher for the dedicated biomass [34]. These present a need to identify more sustainable feedstock options for commercial CNF packaging production, providing environmental neutrality while maintaining the economic benefits [23].

**Figure 3:**
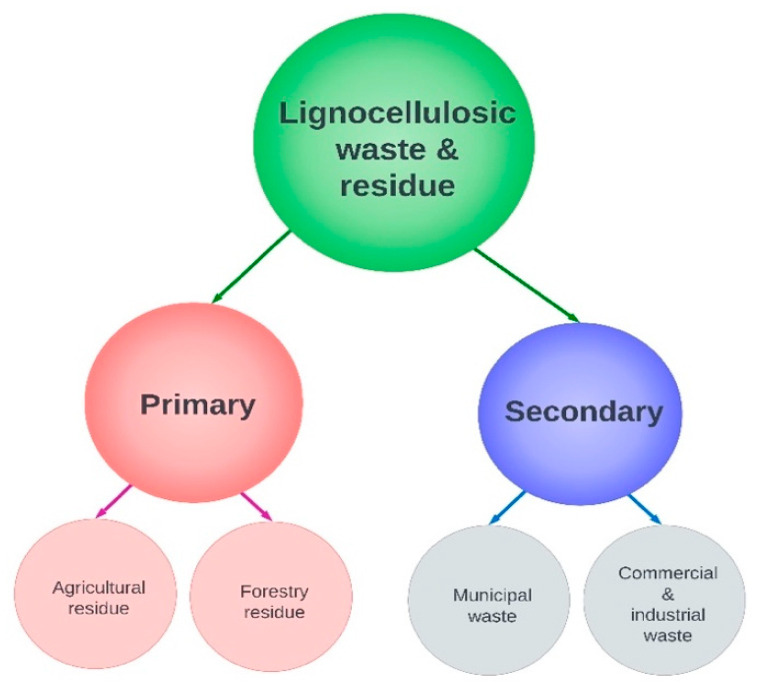
Typology of lignocellulosic wastes.

Compared to dedicated LC, the use of lignocellulosic wastes, i.e., the leftover and eliminated substances of primary processes and applications, lowers the feedstock price and removes the need for land use changes, while producing CNFs with similar properties [37,38]. These wastes— comprising primary residues from forestry (e.g., bark, branches, stump) and agriculture (straw) as well as secondary wastes from municipality, businesses and industries (e.g., waste paper, saw dust, and waste food)— are collectively known as lignocellulosic waste and residue (LCW&R) (See Figure 3) [22,39]. CNFs can also be extracted from algae, bacteria and some animals (e.g., tunicates); however, this study focuses on the readily available, carbon neutral and low-cost feedstock alternative LCW&R [7,23,26].

Many of the LCW&R options either have alternative uses, e.g., straw use in power generation, compost media, and animal bedding, or they go through different end-of-life treatments, e.g., incineration and landfilling of paper wastes [40,41]. Diversion of these materials from current uses and treatments to CNF packaging production requires an evaluation of their economic viability and emission mitigation efficacy [33,34,40]. In addition, it is also imperative to assess the feedstock technical characteristics, i.e., physical and chemical attributes that largely influence the properties and processing requirements for CNF-based packaging [42,43,44].

Existing studies in the literature discuss the impact of various feedstock criteria on CNF film properties, processing requirements and overall LC-based supply chains [20,24,42,45,46,47]. Shanmugam et al. [23] and Ang et al. [29] investigated how the mechanical properties, barrier and recycling performance of CNFs differ for the processed (i.e., dried) and virgin (i.e., never dried) LC. The impact of cell wall structure and composition (e.g., lignin, hemicellulose) of the LC on the CNF properties and process energy consumption were also examined by many authors [19,46,48,49,50]. Existing studies also relate the CNF production yield to the raw material carbohydrate composition [49,51], whereas for industrial LC processing, several studies [20,34,52,53] indicate the influence of feedstock physical properties (e.g., bulk density, durability) and price on the overall production cost. The impact of biomass supply chains on environment, soil and biodiversity were also widely analysed [35,40,54,55]. However, a study consolidating all the above criteria, defining the sustainability of LC wastes for large-scale CNF production, is still absent in the literature.

This study aims to coalesce the sustainability criteria, incorporating technical, economic, and environmental aspects of LCW&R for large-scale CNF packaging production. To this end, we adopted an iterative literature review and expert interviews, and identify thirteen relevant attributes. To demonstrate the use of this criteria pool, we collected the data on LCW&R streams in the UK and analysed how they perform across the given criteria. This helps in better understanding of their sustainability potential in their use for CNF packaging. The approach could be applied to various scenarios to support sustainable feedstock selection for bioplastic packaging and waste management decisions.

## 2. Materials and Methods

An iterative review of relevant academic and grey literature (e.g., reports, briefs and websites) followed by experts’ interviews were conducted to identify the technical, economic and environmental criteria, defining the sustainability of LCW&R for the CNF packaging production. A total of thirteen criteria were identified, categorised as: positive/beneficial, whose higher values are desired; and negative/non-beneficial which were to be as low as possible. Availability (C1), physical composition (C2), cellulose content (C3), hemicellulose content (C4) and bulk density (C5) form the positive criteria, whereas the negative criteria are comprised of lignin content (C6), ash content (C7), cell wall thickness (C8), price of feedstock (C9), seasonal variability (C10), particle size (C11), environmental emission (C12), and soil and biodiversity impact (C13). These criteria are discussed in Section 3.

To demonstrate the waste feedstock sustainability evaluation based on these criteria, data for the current LCW&R streams (See Table 1, Table 2 and Table 3) were collated for the UK. While most of these criteria are objective and measurable that include C1, C3, C4, C5, C6, C7, C8, C9 and C11, others, i.e., C2, C10, C12, C13, are subjective. The quantitative values of the objective attributes are gathered from the existing sources while subjective criteria values are approximated using categorial scales shown in Table 4. The criteria values are then transformed into a coherent quantitative matrix, assessing the sustainability of different waste streams for CNF film production. The criteria values and the sustainability measuring matrix are shown in Section 3. Results. The criteria analysis for the UK waste streams are discussed below.

### 2.1. LCW&R Streams in the UK

In this paper the LCW&R streams refer to the flows of specific LC wastes to various end applications or treatments [56]. To indicate an LCW&R stream, we use the name of the waste material and their existing end use/treatment with a graphical rightward arrow (→) in between, demonstrating the conversion direction (See Table 1, Table 2 and Table 3). Including end uses and treatments within the feedstock options helps to consider their differing environmental impacts as a criterion for feedstock selection. For example, diversion to CNF packaging from two waste streams—‘Wheat straw → Animal bedding’ and ‘Wheat straw → Heat & power’—ought to result in different emission mitigations due to different end uses at present, though both comprise the same material (i.e., wheat straw). A total of 28 LCW&R streams were considered in this study and are denoted with alphanumeric code F1–F28 for the ease of the readers. The LCW&R streams in the UK under primary and secondary categories are discussed below.

#### 2.1.1. Primary Agriculture Residues

The primary agriculture residues are comprised of crop stem, leaves, dead shoots and chaff; for simplification we use the term “straw” to generically denote the residues remaining after extracting grains. Straw is the second largest food supply chain waste and the cost of collection is relatively high [54]. In the UK, the major produced crops are: wheat, barley, oat and oilseed rape [40]. Residues from these crops have many alternative uses that compete and influence the uncertainty of their availability and price [57]. Even when they are not collected for specific applications, they house small insects and return nutrients to the soil [41].

Wheat straw, being less palatable, contributes a small fraction to ‘animal feed’ while the main uses are: ‘animal bedding’, ‘heat and power’, and ‘mushroom and carrot production’ [40,54,57]. Barley and oat straws, being highly nutritious, are mostly used in ‘animal feed’ with a small portion going to ‘animal bedding’, owing to higher price [54]. Oilseed rape straws are brittle and not ideal for ‘animal bedding’ but are increasingly being used for bioenergy, i.e., ‘heat and power’. A proportion of all crop residues are left or chopped and ploughed back into the land, broadly considered here as ‘soil incorporation’. Combining the four crop types with alternative uses, a total of 13 LCW&R streams were identified for the UK primary agriculture residues as shown in Table 1.

#### 2.1.2. Primary Forest Residues

Forest residues are the mix of tree remains, i.e., bark, tops, branches, distorted wood, and in some cases stumps that are left after harvesting [40]. This biomass is expensive to collect and transport [41]. Moreover, extensive collection can cause soil erosion and risks to biodiversity [58].

Forest residues derive from two types of wood: hardwood that comes from broadleaved trees, such as oak, ash and beech; and softwood produced by coniferous trees, e.g., pine, fir, spruce and larch. Considering two material types (conifers, broadleaves) with two end applications (uncollected, heat and power), a total of four LCW&R streams were identified for the UK primary forest residues as shown in Table 2.

#### 2.1.3. Secondary Municipal and Industrial Waste

The secondary LCW&R derive from the lignocellulosic municipal and industrial wastes. The three material groups presenting this waste category are: paper and cardboard, wood and organic. Unlike the primary residues of homogenous materials, secondary LC wastes are mostly comprised of processed and mixed material. Paper and cardboard waste includes paper and card packaging from businesses and households as well as sludges and rejects from the pulp and paper industries [59], whereas wooden packaging, saw dust, bark, chips and cuttings from these industries make up the wood waste. The key components of organic waste come from green and food wastes [59].

The waste treatment routes were identified from the 2018 UK national statistics [60]. The major paper and cardboard wastes go to ‘recycling and reuse’ which is followed by ‘incineration with/out recovery’ and ‘landfilling’. Wood wastes also follow the same treatment routes although ‘backfilling’ is performed to some extent. A major portion of the organic wastes go to organic recycling, i.e., ‘composting and anaerobic digestion’. The LCW&R streams for secondary municipal and industrial wastes are presented in Table 3.

### 2.2. LCW&R Streams Criteria Data Compilation

The positive and negative criteria data for the current LCW&R streams in the UK are gathered in Section 3. The data collection approach used for objective and subjective data are discussed below.

#### 2.2.1. Objective Criteria Data

The quantitative values of objective criteria are gathered from the existing literature except for availability (C1) that is estimated based on both the literature and recent statistics as follows:

Availability of primary agriculture residues: The data on the UK straw availability are not reported, though the crop production data is publicly available [61]. To estimate the current amounts of dry crop residue in the UK, 2021 data on crop areas, yields, moisture content and harvest indices (i.e., the proportion of total dry crop biomass harvested as grain) were used [61,62,63,64]. The proportions of various straws’ applications were then determined based on public datasets and the existing literature [40,54,65,66].

Availability of primary forest residues: Forest residues are not part of the UK national statistics. This study estimated the current dry wood residue biomass for the year 2021 from known forestry statistics [67,68] with the assumptions of harvest site area, wood density, moisture content and the ratio of harvest residues [40,41]. About 50% of the forest residues were considered “uncollected” to comply with the sustainable and good management practices, e.g., ensuring soil cover or adding organic fertilizers [41]. The only application identified for rest of the biomass (collected) was the production of “heat and power” through domestic and industrial combustion [41].

Availability of secondary municipal and industrial waste: To devise the secondary LCW&R streams, generation and treatments of non-hazardous municipal and industrial LC-based wastes in the UK 3454 considered. The latest dataset reporting this information derives from 2018 UK waste statistics [60]. No moisture content was assumed for paper and wood waste, though 82.5% moisture was considered for organic waste [69]. Waste statistics for later years were not used due to being incomplete, and not reflecting the standard waste management practices due to COVID-19.

#### 2.2.2. Subjective Criteria Data

The subjective criteria values shown in Section 3 are defined by various terms based upon the literature and authors’ perception. The four subjective criteria considered in this study are discussed below.

Physical composition: Four subjective ratings were used to define physical composition (C2) (See Section 3). The term ‘raw and homogenous’ is used for all the primary residues from forestry and agriculture. The other three ratings are used for the secondary waste streams: ‘raw and mixed’ for organic; ‘processed and mixed’ for paper; and an intermediate category between these two ‘raw & mixed to processed & mixed’ for wood wastes which were comprised of both processed and unprocessed materials.

Seasonal variability: Seasonal variability (C10), comprising three ratings (high, medium and low) defines three levels of uncertainty associated with the potential availability of the biomass (See Section 3).

Environmental emission: To gauge the change in environmental emission (C12) for feedstock diversion from current practices to CNF packaging, we used the EU Waste Hierarchy, i.e., an order of preference for waste management based on their environmental impact [70]. In this hierarchy, bioplastic production falls in the third step, i.e., reuse, recycling and composting [70]. The waste currently flowing to the treatments below the third step, i.e., energy recovery (i.e., combustion, incineration) and disposal (incineration, landfilling) are considered ‘decrease’ emission when diverted to bioplastic (i.e., CNF) production. The current practices that are likely to involve less processing and chemical use (e.g., feed and bedding material production) are considered ‘increase’ emissions when moved to CNF production [71]. All types of soil incorporation and composting are considered ‘unchanged’ emissions as the CNF end-of-life treatment can take the same route (See Section 3).

Soil and biodiversity impact: Soil and biodiversity impact (C13) only applies to the primary biomass extracted from nature. The primary residue, going to the soil, were considered ‘yes’ (i.e., having an impact on soil and biodiversity) for C13 when moved to CNF films production. The rest of the material streams were considered ‘no’ for C13 (See Section 3).

### 2.3. LCW&R Performance Matrix

Simple calculations are performed to devise the LCW&R performance matrix (See Section 3). All quantitative criteria values were converted to discrete numbers by taking the average if they are expressed as a range. The subjective attributes presented via qualitative data were approximated in discrete quantitative values using the categorial scales shown in Table 4. The data were then normalised to dimensionless indicators in a coherent scale of 0 to 1, using a technique described in the literature [72,73]. The values are presented via data bars in green and red colours for positive and negative criteria, respectively (See Section 3).

## 3. Results

**Figure 4:**
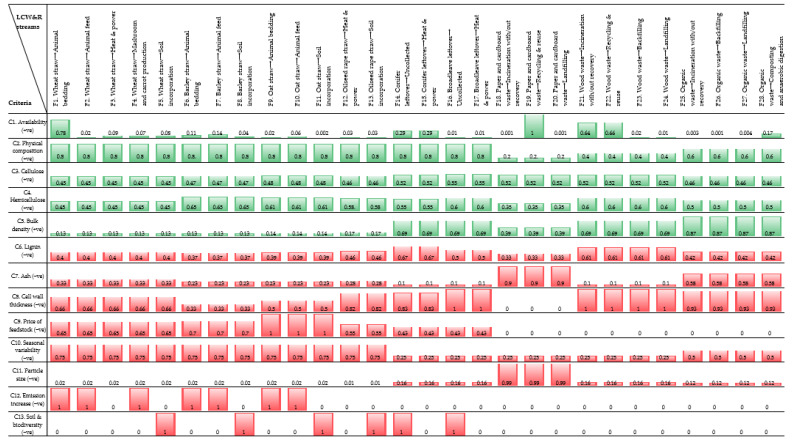
Performance matrix for the UK LCW&R streams. (+ve) and (−ve) are used to indicate positive and negative criteria, respectively.

This section presents the thirteen criteria of LCW&R comprising technical, economic and environmental aspects that collectively determine the feedstock sustainability for CNF packaging production. The criteria values were collated for the 28 LC-waste streams in the UK, as shown with the units of measurements in Table 5 and Table 6. This was converted into a quantitative matrix in Figure 4, mapping sustainability performance of the waste streams along the criteria between 0 to 1.

### 3.1. Sustainability Criteria

The criteria, evaluating the sustainability of using LCW&R from their current practices to the CNF packaging production, are discussed below.

#### 3.1.1. Availability (C1)

Feedstock availability refers to the maximum amount of LCW&R at hand for the CNF packaging production [34,40,74,75]. Knowing the material quantity flowing to various applications/processes at a given time helps to identify which LCW&R stream diversion can achieve economies of scale in the packaging production. Lack of consideration of the availability criteria may cause overstretch or underutilization of the waste material [76]. In the UK (Table 5), F19, i.e., paper and cardboard waste flowing to the recycling operations, presents the overall highest availability, although wheat straws from livestock bedding (F1) enables the maximum feedstock accessibility if primary residues are concerned.

#### 3.1.2. Physical Composition (C2)

This criterion indicates whether an LC stream is comprised of raw, processed, homogenous or mixed materials, determining its requirements for handling or processing operations and the resulting bioplastic quality [77]. Refined biomass is different by chemical composition and processing history than its raw counterpart, and therefore results in CNF films differing in properties, processability or performance [28,29,46]. For example, recycled pulp (i.e., dried once), contrasting to virgin pulp (i.e., never-dried), produces CNF films with reduced tensile strength and swelling capacity, thereby reducing recyclability [28,48,78]. Whereas mixed wastes, e.g., food and garden waste in MSW, possessing heterogenous compositions may cause high costs, requiring more flexible and complex processing in the biorefineries, compared to their homogeneous fractions deriving from forestry and agriculture [79,80,81]. In the UK (Table 5), the primary waste streams (F1–F15) are likely to produce packaging films with better strength and recyclability, albeit using less processing compared to the processed and mixed wastes from flows F16–F28. The use of processed or refined biomass might be restricted in specific cases—such as for food packaging—since regulatory requirements do not allow the use of processed material due to containing harmful chemicals [82].

#### 3.1.3. Cellulose (C3)

Cellulose is the main structural polysaccharide of LC cell walls, that consists of a linear chain of β (1→4)-linked d-glucose units. CNF is partially degraded cellulose with diameters in nanometre scales [17,83,84]. Therefore, the higher the cellulose content in a waste material, the greater the biomass-to-CNF yield. Cellulose has a high degree of polymerisation (DP), and high DP results in better tensile strength for the CNF sheets [85,86,87]. As is seen from Table 5 for the UK, wood residue streams (F14–F17), possessing more cellulose than non-wood residues (F1–F13), which ought to result in better yield and film strength.

#### 3.1.4. Hemicellulose (C4)

Hemicellulose, the second major component of the cell wall, surrounds the cellulose microfibril bundles [83]. Hemicelluloses are branched polysaccharides, containing β-(1→4)-linked backbones of glucose, mannose or xylose in an equatorial configuration [88]. The carboxyl groups in hemicellulose, by the means of electrostatic repulsion, facilitate fibre delamination, reducing fibrillation energy and increasing biomass-to-CNF yields [49]. Additionally, entrenched around cellulose microfibrils with hydrogen bonds, hemicellulose seals the fibril gap and hinders fibril aggregation upon drying, resulting in enhanced film recyclability and cost-effective transportation [19,29,49,50]. The presence of hemicellulose also enhances film properties, e.g., strength and transparency [24,50,89]. Therefore, wood residue and waste from LC streams (F14–F17 and F21–24) in Table 5 (UK scenario), due to higher hemicellulose, should provide better CNF strength and higher production yield than their derivatives, i.e., paper and cardboard in F18–F20.

#### 3.1.5. Bulk Density (C5)

Feedstock delivery cost accounts for 30–35% of the overall costs of an LC supply chain [90]. For cost-effective supply chain, bulk density, i.e., the amount of biomass fitting inside a cubic foot of space, plays a major role [91]. In essence, the greater the bulk density of a biomass, the less space it requires for transportation, handling and storage. Higher density materials require fewer vehicles, as more weight can be placed on each vehicle, reducing the cost of transportation. As is seen from Table 5 for the UK, supply chain costs for agricultural residues derived from F1–F13 is expected to be high, owing to their relatively lower bulk density.

#### 3.1.6. Lignin (C6)

Lignin, a heterogeneous and irregular cross-linked polymer of phenyl propane, binds to cellulose microfibrils in the biomass cell wall [83,92]. With the complex structure, lignin causes biomass recalcitrance to chemical degradation, and restricts CNF extraction [22,83]. Therefore, biomass pre-treatment is performed to remove lignin. The success of the pre-treatment relies on maximum delignification with minimum cellulose loss. Hence, lower lignin composition indicates faster biomass delignification, lesser cellulose loss and lower temperature and chemical use, thereby providing reduced processing costs and energy [20,34]. Therefore, to reduce cost and energy of delignification, paper and organic waste in F18–F20 and F25–F28 (Table 6), with lower lignin contents, are preferred for the UK.

#### 3.1.7. Ash (C7)

Ash refers to the biomass inorganic constituents, e.g., salts of nitrogen, potassium, magnesium, phosphorus, calcium, sulphur, zinc and silicon. Ash rises as the biomass storage period increases, and hence higher ash indicates less durable biomass [93]. Increased ash reduces biomass delignification efficacy, and leads to the wear of mechanical components, e.g., centrifugal pump heads and homogenisation valves [86,94]. During large-scale CNF production, major costs and environmental impacts derive from handling, transportation and disposal of residual ash [85,95]. To illustrate, the lower ash fraction of wood residue and waste (F14–F17 and F21–24) shown in Table 6, is an indication of reduced cost and environmental impact for the wood-based packaging supply chain in the UK.

#### 3.1.8. Particle Size (C8)

Biomass particle size affects its processability and input consumption during the CNF production process [87]. A smaller biomass particle size provides increased specific surface area (surface area of per unit mass) that reduces processing time, and chemical and energy consumption [96]. Decrease in particle size also increases biomass bulk density, reducing the cost of handling and transportation [81,97]. Therefore, size reduction is recommended before transporting the LC to the processing sites [98,99]. In Table 6 for the UK context, we consider biomass as a bulk solid except for paper and cardboard waste, and particle size data were collected from the literature. All agricultural residues (F1–F13) regarded as the finest particles (chopped in 2.42–4.22 mm) ought to consume the least processing time and inputs for CNF packaging production.

#### 3.1.9. Cell Wall Thickness (C9)

High cell wall thickness increases biomass recalcitrance and delays mechanical disintegration, increasing energy consumption [28,49]. Studies [28,46] report that softwood, with a relatively lower cell wall thickness, requires less mechanical treatment than hardwood to produce the equivalent fibrillation level. This observation also applies to non-wood plants; for example, sunflower plants with thinner cell walls takes less fibrillation time compared to alfa, i.e., Stipa tenacissima [49]. As is seen from Table 6, waste paper and cardboard (F18–F20) are considered to have no rigid cell wall, thereby consuming less mechanical energy in CNF packaging production compared to their precursor, i.e., wood (F14–F17 and F21–24) with stiff cell walls.

#### 3.1.10. Price (C10)

Feedstock price is an important and sensitive cost component in the biomass production [34]. High feedstock price acts as a barrier against large-scale development [55]. The price of biomass consists of the costs of labour, energy and machineries that can vary based on location, season and demand [54,66,90]. In the UK, the price of municipal and industrial wastes (F18–F28) is almost zero, making them more cost effective compared to primary forestry and agricultural residues, i.e., F1–F17 (Table 6).

#### 3.1.11. Seasonal Variability (C11)

A major fraction of biomass supply chain costs originates from storage operation, characterized by seasonal variability of the biomass supply [100]. For example, in the UK, the year-round supply of primary forestry residue is possible with small storage operations [40]. However, supply of agricultural residues is highly prone to seasonal uncertainty as straw is collected in a narrow window [40]. The LC composition of municipal food and garden waste is also influenced by the seasonal variation, leading to the requirements for specific storage conditions [101]. Aligning with these notions, the seasonal variability of wood residues and wastes (F14–F17 and F21–24) is regarded as ‘low’, while high seasonal variability is considered for agricultural residues (F1–F13) and so forth (Table 6).

#### 3.1.12. Environmental Emission (C12)

This criterion indicates whether the relocation of LCW&R use to the CNF packaging production would increase, decrease or have no impact on emissions. To understand the emission change, the EU Waste Hierarchy was used as described in Section 2.2 [70]. Thus, in Table 6, diversion of wastes from F3, F15, F17, F18, F20–21, F24–25 and F27 to bioplastic production will ‘decrease’ emissions, whereas, F1–2, F4, F6, F7, F9, and F10 ought to result in emission increase. The rest of the material that are left or used in soil, are considered to result in no emissions change. For enhanced understanding of the relative emissions, a consequential life cycle assessment (LCA) can be adopted [35].

#### 3.1.13. Soil and Biodiversity Impact (C13)

Harvesting primary residues can have significant impacts on soil and biodiversity. These residues are considered as important habitats for microorganisms, fungi, insects, and birds [58,102]. Excessive extraction of forest and agricultural biomass can reduce soil productivity, moisture retention and aeration [102,103]. To comply with the sustainable harvesting guidelines, limited extraction is performed in many countries; however, these rules do not constrain secondary waste use [40,58]. The residue portions that are left or intended for land incorporation (F5, F8, F11, F13, F14, F16 in Table 6 for the UK) will have an impact on soil and biodiversity if collected to produce CNF packaging. However, the waste streams already collected for various applications do not cause these impacts.

### 3.2. LCW&R Performance Matrix

Table 5 and Table 6 is combined and converted into a performance matrix in Figure 4, evaluating how each LCW&R stream performs across the proposed criteria for the UK context. The normalised scores are shown via green and red data bars for the beneficial and nonbeneficial criteria, respectively.

Paper and cardboard wastes for recycling (F19) provide the highest feedstock availability (C1), with no increased emissions (C12) or soil and biodiversity impact (C13), although they may result in lack of film properties due to low hemicellulose (C4) and lacking in physical composition (C2). Among the waste streams with a higher C2 level, i.e., raw and homogenous, wheat straw from livestock bedding (F1) tops in availability (C1), although it will increase emissions (C12) when moved to CNF packaging production. The yield and mechanical properties are expected to be the highest for wood residues and wastes (F14–17 and F21–24) owing to their maximum cellulose (C3) and hemicellulose (C4) compositions, yet they will consume more energy in CNF processing due to the highest lignin content (C6) and cell wall thickness (C8). Among these wood streams, extraction of the uncollected residues (F14, F16) may increase the soil and biodiversity impact (C13). The secondary waste streams treated in incineration and landfilling (F18, F21, F25, F27), come at an almost negligible feedstock price (C9) and do not increase emissions (C12) or soil and biodiversity impacts (C13); nevertheless, they may increase the processing cost, being characterised with processed and mixed materials (C2).

**Table 5:** Positive criteria (C1–C5) values for LCW&R streams in the UK.

	Criteria	C1. Availability (Dry Tonnes)	C2. Physical Composition (Subjective)	C3. Cellulose (wt%)	C4. Hemicellulose (wt%)	C5. Bulk Density (kg/m^3^)
LCW&R						
F1. Wheat straw → Animal bedding		3073851.20	Raw & homogenous	33–40 [40]	20–25 [40]	36.22–39.74 [81]
F2. Wheat straw → Animal feed		81534.52	Raw & homogenous	33–40 [40]	20–25 [40]	36.22–39.74 [81]
F3. Wheat straw → Heat & power		364008.70	Raw & homogenous	33–40 [40]	20–25 [40]	36.22–39.74 [81]
F4. Wheat straw → Mushroom and carrot production		278933.87	Raw & homogenous	33–40 [40]	20–25 [40]	36.22–39.74 [81]
F5. Wheat straw → Soil incorporation		314048.90	Raw & homogenous	33–40 [40]	20–25 [40]	36.22–39.74 [81]
F6. Barley straw → Animal bedding		433491.83	Raw & homogenous	31–45 [40]	27–38 [40]	33.89–38.61 [81]
F7. Barley straw → Animal feed		542612.20	Raw & homogenous	31–45 [40]	27–38 [40]	33.89–38.61 [81]
F8. Barley straw → Soil incorporation		149533.75	Raw & homogenous	31–45 [40]	27–38 [40]	33.89–38.61 [81]
F9. Oat straw → Animal bedding		61739.75	Raw & homogenous	31–48 [40]	23–38 [40]	38.61–41.69 [81]
F10. Oat straw → Animal feed		227798.37	Raw & homogenous	31–48 [40]	23–38 [40]	38.61–41.69 [81]
F11. Oat straw → Soil incorporation		6052.35	Raw & homogenous	31–48 [40]	23–38 [40]	38.61–41.69 [81]
F12. Oilseed rape straw → Heat & power		133469.86	Raw & homogenous	35–40 [40]	27–31 [40]	47.46–49.7 [81]
F13. Oilseed rape straw → Soil incorporation		106883.32	Raw & homogenous	35–40 [40]	27–31 [40]	47.46–49.7 [81]
F14. Conifer leftovers → Uncollected		1156979	Raw & homogenous	35–45 [40]	25–30 [40]	128–267 [104]
F15. Conifer leftovers → Heat & power		1156979	Raw & homogenous	35–45 [40]	25–30 [40]	128–267 [104]
F16. Broadleaf leftovers → Uncollected		22231	Raw & homogenous	40–50 [40]	25–35 [40]	128–267 [104]
F17. Broadleaf leftovers → Heat & power		22231	Raw & homogenous	40–50 [40]	25–35 [40]	128–267 [104]
F18. Paper and cardboard waste → Incineration with/out recovery		3811.08	Processed & mixed	40–50 [105,106]	0–35 [105,106]	112 [107,108]
F19. Paper and cardboard waste → Recycling & reuse		3936954.05	Processed & mixed	40–50 [105,106]	0–35 [105,106]	112 [107,108]
F20. Paper and cardboard waste → Landfilling		5062.33	Processed & mixed	40–50 [105,106]	0–35 [105,106]	112 [107,108]
F21. Wood waste → Incineration with/out recovery		2536972.89	Raw & homogeneous to processed & mixed	40–50 [40]	25–35 [40]	128–267 [104]
F22. Wood waste → Recycling & reuse		2600381.03	Raw & homogeneous to processed & mixed	40–50 [40]	25–35 [40]	128–267 [104]
F23. Wood waste → Backfilling		88781.00	Raw & homogeneous to processed & mixed	40–50 [40]	25–35 [40]	128–267 [104]
F24. Wood waste → Landfilling		22185.97	Raw & mixed to processed & mixed	40–50 [40]	25–35 [40]	128–267 [104]
F25. Organic waste → Incineration with/out recovery		13246.16	Raw & mixed	25.7–55.4 [40,109]	7.2–43 [40,109]	200–300 [110]
F26. Organic waste → Backfilling		2058	Raw & mixed	25.7–55.4 [40,109]	7.2–43 [40,109]	200–300 [110]
F27. Organic waste → Landfilling		14452.29	Raw & mixed	25.7–55.4 [40,109]	7.2–43 [40,109]	200–300 [110]
F28. Organic waste → Composting and anaerobic digestion		682814.19	Raw & mixed	25.7–55.4 [40,109]	7.2–43 [40,109]	200–300 [110]

**Table 6:** Negative criteria (C6–C13) values for LCW&R streams in the UK.

	Criteria	C6. Lignin (wt%)	C7. Ash (wt%)	C8. Cell Wall Thickness (µm)	C9. Price of Feedstock (£/tonne)	C10. Seasonal Variability (Subjective)	C11. Particle Size (mm)	C12. Environmental Emission (Subjective)	C13. Soil and Biodiversity Impact (Subjective)
LCW&R									
F1. Wheat straw → Animal bedding		15–21 [40]	3–10 [40]	3.96 [111]	39–105 [112]	High	4.22 (chopped) [81]	Increase	No
F2. Wheat straw → Animal feed		15–21 [40]	3–10 [40]	3.96 [111]	39–105 [112]	High	4.22 (chopped) [81]	Increase	No
F3. Wheat straw → Heat & power		15–21 [40]	3–10 [40]	3.96 [111]	39–105 [112]	High	4.22 (chopped) [81]	Decrease	No
F4. Wheat straw → Mushroom and carrot production		15–21 [40]	3–10 [40]	3.96 [111]	39–105 [112]	High	4.22 (chopped) [81]	Increase	No
F5. Wheat straw → Soil incorporation		15–21 [40]	3–10 [40]	3.96 [111]	39–105 [112]	High	4.22 (chopped) [81]	Unchanged	Yes
F6. Barley straw → Animal bedding		14–19 [40]	2–7 [40]	up to 2 [113]	45–108 [112]	High	3.37 (chopped) [81]	Increase	No
F7. Barley straw → Animal feed		14–19 [40]	2–7 [40]	up to 2 [113]	45–108 [112]	High	3.37 (chopped) [81]	Increase	No
F8. Barley straw → Soil incorporation		14–19 [40]	2–7 [40]	Up to 2 [113]	45–108 [112]	High	3.37 (chopped) [81]	Unchanged	Yes
F9. Oat straw → Animal bedding		16–19 [40]	2–7 [40]	2–3.96 [114]	50–170 [112]	High	4.15 (chopped) [81]	Increase	No
F10. Oat straw → Animal feed		16–19 [40]	2–7 [40]	2–3.96 [114]	50–170 [112]	High	4.15 (chopped) [81]	Increase	No
F11. Oat straw → Soil incorporation		16–19 [40]	2–7 [40]	2–3.96 [114]	50–170 [112]	High	4.15 (chopped) [81]	Unchanged	Yes
F12. Oilseed rape straw → Heat & power		18–23 [40]	3–8 [40]	4.91 [115]	41–80 [112]	High	2.42 (chopped) [81]	Decrease	No
F13. Oilseed rape straw → Soil incorporation		18–23 [40]	3–8 [40]	4.91 [115]	41–80 [112]	High	2.42 (chopped) [81]	Unchanged	Yes
F14. Conifer leftover → Uncollected		25–35 [40]	1–3 [40]	2–8 [116]	35–60 [55]	Low	0–63 (chipped) [104]	Unchanged	Yes
F15. Conifer leftover → Heat & power		20–25 [40]	1–3 [40]	2–8 [116]	35–60 [55]	Low	0–63 (chipped) [104]	Decrease	No
F16. Broadleaf leftovers → Uncollected		20–25 [40]	1–3 [40]	1–11 [117]	35–60 [55]	Low	0–63 (chipped) [104]	Unchanged	Yes
F17. Broadleaf leftovers → Heat & power		0–30 [105,106]	1–3 [40]	1–11 [117]	35–60 [55]	Low	0–63 (chipped) [104]	Decrease	No
F18. Paper and cardboard waste → Incineration with/out recovery		0–30 [105,106]	0–35 [118,119]	Not applicable	Negligible [40]	Low	100–300 (baled) [107]	Decrease	No
F19. Paper and cardboard waste → Recycling & reuse		0–30 [105,106]	0–35 [118,119]	Not applicable	Negligible [40]	Low	100–300 (baled) [107]	Unchanged	No
F20. Paper and cardboard waste → Landfilling		0–30 [105,106]	0–35 [118, 119]	Not applicable	Negligible [40]	Low	100–300 (baled) [107]	Decrease	No
F21. Wood waste → Incineration with/out recovery		20–35 [40]	1.0–3.0 [40]	1–11 [116,117]	Negligible [40]	Low	0–63 (chipped) [104]	Decrease	No
F22. Wood waste → Recycling & reuse		20–35 [40]	1.0–3.0 [40]	1–11 [116,117]	Negligible [40]	Low	0–63 (chipped) [104]	Unchanged	No
F23. Wood waste → Backfilling		20–35 [40]	1.0–3.0 (Used same as forest residues) [40]	1–11 [116,117]	Negligible [40]	Low	0–63 (chipped) [104]	Unchanged	No
F24. Wood waste → Landfilling		3–35 [40]	1.0–3.0 (Used same as forest residues) [40]	1–11 [116,117]	Negligible [40]	Low	0–63 (chipped) [104]	Decrease	No
F25. Organic waste → Incineration with/out recovery		3–35 [40]	2.5–20 [120,121]	0.1–11 [122]	Negligible [40]	Medium to High	10–40 (shredded) [110]	Decrease	No
F26. Organic waste → Backfilling		25–35 [40]	2.5–20 [120,121]	0.1–11 [122]	Negligible [40]	Medium to High	10–40 (shredded) [110]	Unchanged	No
F27. Organic waste → Landfilling		3–35 [40]	2.5–20 [120,121]	0.1–11 [122]	Negligible [40]	Medium to High	10–40 (shredded) [110]	Decrease	No
F28. Organic waste → Composting and anaerobic digestion		3–35 [40]	2.5–20 [120,121]	0.1–11 [122]	Negligible [40]	Medium to High	10–40 (shredded) [110]	Unchanged	No

## 4. Discussion

Sustainability assessment of LCW&R for CNF packaging production is a complex problem, requiring combinatorial consideration of technical, economic and environmental aspects [33,34,43]. These characteristics are mentioned disjointly across the literature [20,24,46,47], and a consolidated overview is absent. This study used an iterative literature review and experts’ interviews, and identified 13 criteria pertaining to LC waste sustainability for CNF packaging production. The criteria list includes: availability, physical composition, cellulose content, hemicellulose content, bulk density, lignin content, ash content, cell wall thickness, price of feedstock, seasonal variability, particle size, environmental emission, and soil and biodiversity impact. These criteria data were collected for the LCW&R streams in the UK (Table 5 and Table 6), and were combined into a coherent matrix to assess their performance (Figure 4). This study helps to uncover the sustainability potential of LCW&R for CNF packaging production, encompassing technical properties as well as environmental and economic criteria.

**Figure 5:**
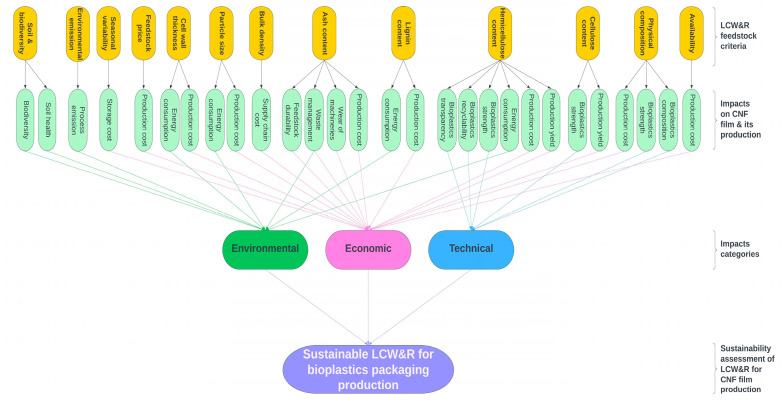
The thirteen feedstock criteria with impacts under technical, economic and environmental categories used for sustainability assessment of LCW&R for CNF packaging production.

Feedstock attributes, influencing the properties and performance of the CNF packaging films, make up the technical criteria. They include physical composition, cellulose and hemicellulose contents that determine the strength, transparency, and recyclability of the packaging films. Most of the feedstock characteristics including availability, physical composition, bulk density, lignin and price, were found to have influence on the economic aspects of CNF packaging production, e.g., production yield, processing and supply chain costs, wear of machineries and raw material storage. Whereas environmental dimensions such as energy consumption, waste management, emission and soil or biodiversity impacts are correlated to certain feedstock criteria, e.g., bulk density, lignin, ash, particle size, emission, soil and biodiversity impact, and so on. Most of the sustainability assessment criteria were found to influence more than one aspect (technical, economic, environmental) of CNF packaging production (see Figure 5).

Consideration of technical, economic and environmental factors under the sustainability umbrella presents a more comprehensive approach to feedstock sustainability assessment compared to the existing literature [24,35,53]. Although existing studies present a combined synopsis of feedstock criteria for other LC derivatives, e.g., biofuel and paper, a review of bioplastic feedstock criteria is a new contribution [40,85,86]. Moreover, the criteria list presented in this paper can be used to assess the existing waste material streams/flows instead of just the material itself, taking into account the environmental impact for replacing the current practices [33].

The LCW&R streams in the UK have been analysed based on the identified criteria and presented through a performance matrix (Table 5 and Table 6 and Figure 4). Paper and cardboard wastes intended for recycling (F19) provides the highest feedstock availability, although they may result in lack of film mechanical properties. The highest mechanical properties can derive from wood residues and wastes (F14–17 and F21–24), but high process energy consumption will be a barrier. Moreover, the extraction of uncollected residues (F14, F16) may cause soil and biodiversity impact, although more fractions could be obtained from designated locations [58]. Diversion of secondary waste streams from incineration and landfilling (F18, F21, F25, F27) will reduce feedstock cost, environmental emissions and soil and biodiversity impacts; however, processing costs may increase due to the presence of heterogenous materials. The analysis technique used in this paper can be adopted in wide range of scenarios, requiring LC waste material diversion from existing uses to the production of CNF products including packaging.

This study explored the basic criteria for assessing sustainability of LC wastes in CNF packaging production. The results of this study will be considered in our forthcoming research on bioplastic feedstock decision analytics. Future research opportunity exists for consolidating empirical results to examine or enhance the proposed criteria. Further criteria distinctions can be considered based on: location, climatic conditions, plant species, crop cultivation, fibre location in plant, fibre age and presence of non-structural components (i.e., extractives). Moreover, the chemical pre-treatments and mechanical fibrillations used can influence the properties of the resulting CNF films, and thus can be integrated with feedstock criteria analysis to identify the overall sustainable routes for commercial CNF packaging production [42].

## 5. Conclusions

This study presents criteria for assessing the sustainability of LCW&R, incorporating technical, economic, and environmental aspects, for large-scale CNF packaging production. Thirteen relevant attributes were identified through an iterative literature review and expert interviews. Further, we gathered the criteria data for the UK waste streams and converted them into a performance matrix to measure the feedstock sustainability. This research will help to identify low-cost feedstocks and design biorefineries and supply chains for CNF packaging, replacing the petrochemical plastics. This study can also help in waste management decisions by identifying the waste material streams for which bioplastic packaging production and environmental emission reduction is complimentary rather than conflicting. This will support the inclusion of bioplastic processing in the national waste management plan, facilitating a circular bioeconomy.

## Figures and Tables

**Table 1:** LCW&R streams for primary agriculture residues.

	Wheat Straw	Barley Straw	Oat Straw	Oilseed Rape Straw
Animal bedding	Wheat straw → Animal bedding (F1)	Barley straw → Animal bedding (F6)	Oat straw → Animal bedding (F9)	—
Animal feed	Wheat straw → Animal feed (F2)	Barley straw → Animal feed (F7)	Oat straw → Animal feed (F10)	—
Heat & power	Wheat straw → Heat & power (F3)	—	—	Oilseed rape straw → Heat & power (F12)
Mushroom and carrot production	Wheat straw → Mushroom and carrot production (F4)	—	—	—
Soil incorporation	Wheat straw → Soil incorporation (F5)	Barley straw → Soil incorporation (F8)	Oat straw → Soil incorporation (F11)	Oilseed rape straw → Soil incorporation (F13)

**Table 2:** LCW&R streams for primary forest residues.

	Conifer Leftover	Broadleaf Leftover
Uncollected	Conifer leftover → Uncollected (F14)	Broadleaf leftover → Uncollected (F16)
Heat & power	Conifer leftover → Heat & power (F15)	Broadleaf leftover → Heat & power (F17)

**Table 3:** LCW&R streams for secondary municipal and industrial wastes.

	Paper and Cardboard Waste	Wood Waste	Organic Waste
Incineration with/out recovery	Paper and cardboard waste → Incineration with/out recovery (F18)	Wood waste → Incineration with/out recovery (F21)	Organic waste → Incineration with/out recovery (F25)
Recycling & reuse	Paper and cardboard waste → Recycling & reuse (F19)	Wood waste → Recycling & reuse (F22)	—
Backfilling	—	Wood waste → Backfilling (F23)	Organic waste → Backfilling (F26)
Landfilling	Paper and cardboard waste → Landfilling (F20)	Wood waste → Landfilling (F24)	Organic waste → Landfilling (F27)
Composting and anaerobic digestion	—	—	Organic waste → Composting and anaerobic digestion (F28)

**Table 4:** Scales used for quantitative estimation of subjective criteria.

Subjective Rating	Quantitative Rating
Physical composition	
Raw & homogenous	4
Raw & mixed	3
Raw & mixed to processed & mixed	2
Processed & mixed	1
Seasonal variability	
High	3
Medium	2
Low	1
Environmental emission	
Increase	1
Decrease or unchanged	0
Soil and biodiversity impact	
Yes	1
No	0

## Data Availability

The data presented in this study are available upon reasonable request from the corresponding author.
